# Influenza A Virus Antibodies in Ducks and Introduction of Highly Pathogenic Influenza A(H5N1) Virus, Tennessee, USA

**DOI:** 10.3201/eid3012.241126

**Published:** 2024-12

**Authors:** David E. Stallknecht, Deborah L. Carter, Abigail G. Blake-Bradshaw, Nicholas M. Masto, Cory J. Highway, Jamie C. Feddersen, Richard Webby, Bradley Cohen, Jeffery D. Sullivan, Rebecca Poulson

**Affiliations:** University of Georgia, Athens, Georgia, USA (D.E. Stallknecht, D.L. Carter, R. Poulson); Tennessee Technical University, Cookeville, Tennessee, USA (A.G. Blake-Bradshaw, C.J. Highway, B. Cohen); Cornell University, Ithaca, New York, USA (N.M. Masto); Tennessee Wildlife Resources Agency, Nashville, Tennessee, USA (J.C. Feddersen); St. Jude Children’s Research Hospital, Memphis, Tennessee, USA **(**R. Webby); US Geological Survey, Laurel, Maryland, USA (J.D. Sullivan)

**Keywords:** avian influenza virus, viruses, ducks, H5N1, highly pathogenic avian influenza, Tennessee, United States

## Abstract

Testing of ducks in Tennessee, United States, before introduction of highly pathogenic influenza A(H5N1) virus demonstrated a high prevalence of antibodies to influenza A virus but very low prevalence of antibodies to H5 (25%) or H5 and N1 (13%) subtypes. Antibody prevalence increased after H5N1 introduction.

Highly pathogenic (HP) influenza A virus (IAV), subtype H5N1, clade 2.3.4.4b was detected in North America in November 2021; many infections and mass deaths were subsequently reported in wild birds from North America to Antarctica (*1*–*3*). The IAV immunologic status in waterfowl before the HP H5N1 introduction are largely unknown; however, infections with endemic low pathogenicity (LP) IAV occur in ducks every year and peak during fall migration (*4*,*5*). Prevalence of IAV antibodies in waterfowl also increases during migration; reported estimates in mallard ducks (*Anas platyrhynchos*) were 51%–75% for hatch year and 86%–93% for *A. platyrhynchos* sampled in September after hatch year (*6*). In contrast, prevalence of infection and antibodies to specific IAV hemagglutinin (HA) and neuraminidase (NA) subtypes, such as H5 and N1, can vary annually and often are low (*4*,*6*). How immunity from previous LP IAV exposure, especially with LP H5 and N1 IAV subtypes, affects duck susceptibility to HP H5N1 infection, illness, and death, or transmission and maintenance of new IAV in the system, have not been adequately explored. Data from field studies and experimental infections of waterfowl supported the need for further study and demonstrated that previous infections with LP IAV can reduce infection, death, and viral shedding during subsequent infections with both LP and HP IAV and increase the infective dose required for infection (*7*–*9*). Those effects are more pronounced with genetically similar or matched HA subtypes and repeated LP IAV infections (*10*,*11*). NA is associated with susceptibility of mice and humans to IAV infection (*12*), but less is known about potential protective effects of immunity to NA in ducks. We sampled ducks to investigate IAV immunity in waterfowl before and after HP H5N1 was detected in Tennessee, USA.

## The Study

We tested cloacal and oropharyngeal swab samples from ducks sampled in western Tennessee, United States, during November 7, 2021–January 31, 2022 for IAV infection before and after detection of HP H5N1 at the study site on January 24, 2022. A telemetry study conducted on a subsample of those birds showed no effect of HP H5N1 infection on survival or movement of mallard ducks (*13*). We collected serum samples (not included in the telemetry study) that enabled us to estimate prevalence of antibodies to IAV nucleoprotein (NP), to IAV HA subtype H5, and to NA subtype N1 before identification of HP H5N1 introduction into the population; document short-term antibody responses in the population after HP H5N1 introduction; examine antibody levels in individual ducks infected with HP H5N1; and address the need to consider population immunity when evaluating effects of HP H5N1 infection on wild avian species. Duck capture and handling procedures in this study were conducted in accordance with Tennessee Technological University’s Institutional Animal Care and Use Committee (IACUC protocol no. 19-20-002) and authorized under Federal Banding Permit nos. 05796 and 24239.

We tested combined cloacal and oropharyngeal swab samples for IAV, including H5 clade 2.3.4.4b, by virus isolation in embryonating chicken eggs and by real-time reverse transcription PCR, as previously described (*13*). We submitted all H5 nonnegative isolates to the US Department of Agriculture National Veterinary Services Laboratory (Ames, Iowa) for confirmation of subtype and pathogenicity. Duck species tested included mallard (n = 236), northern pintail (*A. acuta*, n = 19), gadwall (*Mareca strepera*, n = 4), and American wigeon (*M. americana*, n = 3). We did not detect HP H5N1 or LP IAV in any samples collected before January 24, 2022 (n = 220). During January 24–31, 2022, the laboratory detected and confirmed HP H5N1 in samples collected from 12/38 (32%) mallards, 3/4 (75%) gadwalls, and 2/2 (100%) American wigeon. Phylogenetic analyses of sequences from a subset of isolated IAV indicated that sequences belonged to genotype A1 of clade 2.3.4.4b viruses, the same genotype as the virus clade first detected in North America in Canada (*3*).

We tested serum samples for antibodies to IAV NP using a commercial bELISA (IDEXX AI MultiS-Screen AB test; IDEXX Laboratories, https://www.idexx.com). We further tested samples testing positive (sample to negative absorbance ratio <0.7) for antibodies againts H5 and N1 by using hemagglutination inhibition (HI), virus neutralization (VN), and enzyme linked lectin assay (ELLA), as described previously (*6*,*14*). We further tested only a subset of samples from the pre–H5N1 detection period. We used 2 attenuated viruses produced by reverse genetics as antigens for HI and VN: reverse genetics BWT, containing HA and NA from the LP A/Blue-winged teal/AI12–4150/Texas/2012 (H5N2), and reverse genetics AST IDCDC-RG71A (H5N8), containing a modified HA and NA from HP 2.3.4.4b A/Astrakhan/3212/2020 (H5N8); remaining gene segments from both viruses were from A/Puerto Rico/8/34. For ELLA, we used A/ruddy turnstone/New Jersey/AI13–2948/2013 (H10N1) as an antigen. We considered samples that tested positive for either antigen at a titer of 32 for HI or 20 for VN positive for H5 antibodies. For ELLA we considered a titer of 80 positive.

IAV NP antibodies were prevalent (79%) in ducks at the study site before introduction of HP H5N1 virus; antibody prevalence increased to 100% after H5N1 was detected (Table 1). Antibody prevalence for H5, based on reactivity to reverse genetics BWT or reverse genetics AST, however, was low before HP H5N1 introduction, as determined by both HI (8%) and VN (25%). By the postdetection sample period, January 30–31, 2022, antibody prevalence increased, as determined by HI (27%) and VN (53%) (Table 1). Antibody prevalence to N1 subtype also increased from 40% before HP H5N1 detection to 86% after detection (Table 1). We observed an increase in the percentage of ducks testing seropositive for H5 after HP H5N1 detection by both HI and VN (Table 2). For H5N1-infected ducks, 15/17 (88%) tested seropositive for NP, 1 (6%) tested seropositive for H5 by HI, 2 (12%) were seropositive for H5 by VN, and 12/16 (75%) tested seropositive for N1. Some of those antibody-positive results, especially in postdetection sampling, could have resulted from seroconversion, but still reflect a very low prevalence of antibodies to H5 at time of infection.

**Table 1:** Antibodies detected in wintering ducks in study of influenza A virus antibodies in ducks and introduction of highly pathogenic influenza A(H5N1) virus, Tennessee, USA*

Time sampled	Total	HI H5†	VN H5†	ELLA N1‡	Antibodies to H5 and N1§	
					Total	Prevalence, %¶
Pre–H5N1 detection, Nov 7, 2021–Jan 5, 2022						
Total	0/218 (0)	6/63 (10)	21/65 (32)	33/65 (51)	11/65 (17)	13
bELISA-positive	173/218 (79)					
** % **Testing positive		8	25	40		
H5N1 detection, Jan 24–25, 2022						
Total	14/29 (48)	1/26 (4)	3/26 (12)	15/24 (63)	3/24 (13)	12
bELISA-positive	26/29 (90)					
** % **Testing positive		4	10	57		
Post–H5N1 detection, Jan 30–31, 2022						
Total	3/15 (20)	4/15 (27)	8/15 (53)	11/13 (85)	8/13 (62)	62
bELISA-positive	15/15 (100)					
** % **Testing positive		27	53	85		

*Values are no. positive/no. tested (%) except as indicated. For HI, VN, and ELLA, testing was limited to bELISA-positive serum. bELISA, blocking enzyme-linked immunosorbent assay; ELLA, enzyme-linked lectin assay; HI, hemagglutination inhibition; VN, virus neutralization. †Tests completed with 2 antigens: reverse genetics BWT antigen, HA and NA from A/Blue-winged teal/AI12–4150/Texas/2012 (H5N2) on PR8 backbone; and reverse genetics AST antigen, IDCDC-RG71A (H5N8) with modified HA and NA from A/Astrakhan/3212/2020 (H5N8) on PR8 backbone. ‡Test used A/ruddy turnstone/New Jersey/AI13–2948/2013(H10N1) as antigen. §Ducks tested positive for antibodies against H5 and N1 as determined by HI, VN, or both, and ELLA. ¶Apparent prevalence in sampled population, adjusted for bELISA results.

**Table 2:** Antibodies to H5 detected in wintering ducks in a study of influenza A virus antibodies in ducks and introduction of highly pathogenic influenza A(H5N1) virus, Tennessee, USA*

Time sampled	Hemagglutination inhibition H5†			Virus neutralization H5†	
	rg BWT	rg AST		rg BWT	rg AST
Pre–H5N1 detection, Nov 7, 2021–Jan 5, 2022	6/63 (10)	1/63 (2)		21/65 (32)	2/65 (3)
H5N1 detection, Jan 24–25, 2022	1/26 (4)	0/26 (0)		3/24 (13)	1/26 (4)
Post–H5N1 detection, Jan 30–31, 2022	4/15 (27)	3/15 (20)		7/12 (58)	3/12 (25)

*Values are no. positive/no. tested (%). As determined by hemagglutination inhibition and virus neutralization using 2 H5 antigens of North American low pathogenicity IAV (reverse genetics BWT) and goose/Guangdong clade 2.3.4.4b H5 lineage of highly pathogenic IAV (reverse genetics AST). AST, IDCDC-RG71A (H5N8) with modified HA and NA from A/Astrakhan/3212/2020 (H5N8) on PR8 backbone; BWT, HA and NA from A/Blue-winged teal/AI12–4150/Texas/2012 (H5N2) on PR8 backbone; HA, hemagglutinin; HI, hemagglutination inhibition; IAV, influenza A virus; NA, neuraminidase; rg, reverse genetics; VN, virus neutralization. †Both tests completed with 2 antigens. Reverse genetics BWT antigen: HA and NA from A/Blue-winged teal/AI12–4150/Texas/2012 (H5N2) on PR8 backbone; and reverse genetics AST antigen: IDCDC-RG71A (H5N8). Modified HA and NA from A/Astrakhan/3212/2020 (H5N8) on PR8 backbone.

## Conclusions

Although antibody prevalence to NP was high in ducks before HP H5N1 virus introduction at the site, likely reflecting previous infections with LP IAV, the prevalence of antibodies to H5 and N1 subtypes was low; few (11 [17%]) ducks tested seropositive for both (Table 1). The lack of immunity to H5 and N1 possibly enabled the successful introduction of HP H5N1 into this population and into North America. That pattern was also apparent in individual ducks that were infected with HP H5N1 at the time we collected serum samples. We detected an increase in antibody prevalence with all serologic tests after HP H5N1 introduction; 62% of birds sampled in the postdetection sample period tested seropositive for both H5 and N1. Whether the developing immunity affected subsequent HP H5N1 transmission as birds migrated from this site is unknown, but many (38%) departing birds apparently remained unexposed. The population was not immunologically naive to IAV at the time of HP H5N1 introduction and could have been partially protected from clinical effects caused by existing heterosubtypic immunity; however, that did not prevent infection or rapid transmission through the population. After HP H5N1 introduction, substantial immunity to H5 and N1 was present. Future studies should address whether existing heterosubtypic or developing homosubtypic immunity affected survival or migration of this duck population; such studies could apply to other wild bird species that may be more vulnerable than ducks to HP H5N1. Monitoring IAV in wild migratory populations can inform influenza spillover risks to domestic animals and humans.
